# Caring Under Pressure: Perceived Stress Among Caregivers of Dementia Patients

**DOI:** 10.1192/j.eurpsy.2025.1593

**Published:** 2025-08-26

**Authors:** O. Inanc

**Affiliations:** 1 Gulhane Training and Research Hospital, Ankara, Türkiye

## Abstract

**Introduction:**

Dementia is a progressive neurodegenerative disorder characterized by a gradual decline in cognitive functions, such as memory, reasoning, and communication, which places a significant burden on caregivers. Dementia caregiving presents unique challenges that contribute to elevated stress levels. Caregivers of dementia patients often face increased psychological and physical strain, exacerbated by the disease’s progressive nature and complex caregiving demands. This systematic review explores the perceived stress among dementia caregivers, highlighting the impact of caregiving responsibilities and identifying potential coping mechanisms. Our review seeks to offer evidence-based recommendations to support caregivers and improve their quality of life, thereby addressing a crucial gap in dementia care.

**Objectives:**

This systematic review aims to assess the extent of perceived stress among dementia caregivers, identify associated factors, and evaluate coping mechanisms. The goal is to highlight critical stressors and propose evidence-based strategies for improving caregiver support.

**Methods:**

We systematically reviewed eighteen studies from PubMed using the terms “Perceived Stress Scale” and “Caregivers of Dementia Patients.” Our analysis included data from 3,385 caregivers, focusing on stress levels, cortisol measurements, and psychological impacts.

**Results:**

The review consistently identified high levels of perceived stress among dementia caregivers. Cortisol levels, assessed in three studies, were significantly elevated in caregivers compared to non-caregivers (Williams et al., 2021; Kim & Park, 2022; Nguyen et al., 2023). Perceived stress showed a significant correlation with cognitive decline and sundowning behaviors. Additionally, caregivers reported elevated depressive symptoms, with stress levels showing a direct association with these symptoms (Jones et al., 2021). The review also identified that higher education was associated with reduced anxiety and depression, while female caregivers reported higher stress levels compared to males (Miller et al., 2022). Effective coping strategies included seeking information, participating in culturally adapted interventions, and enhancing social support networks (Patel & Singh, 2023).

**Conclusions:**

Dementia caregiving imposes considerable stress due to the demanding nature of the role and the complexities of patients’ conditions. Interventions should prioritize culturally sensitive support programs, expand educational opportunities for caregivers, and strengthen social support systems to alleviate caregiver stress. Future research should further investigate the influence of gender, social isolation, and healthcare access on caregiver stress, particularly in low- and middle-income countries (LMICs) and during global crises such as the COVID-19 pandemic. Addressing these factors will be crucial in developing comprehensive strategies to support dementia caregivers effectively.

**Disclosure of Interest:**

None Declared

